# Characterizing the Expression Patterns of Parkinson’s Disease Associated Genes

**DOI:** 10.3389/fnins.2021.629156

**Published:** 2021-04-01

**Authors:** Bin Li, Guihu Zhao, Kuokuo Li, Zheng Wang, Zhenghuan Fang, Xiaomeng Wang, Tengfei Luo, Yi Zhang, Yijing Wang, Qian Chen, Yuanfeng Huang, Lijie Dong, Jifeng Guo, Beisha Tang, Jinchen Li

**Affiliations:** ^1^Department of Geriatrics, National Clinical Research Center for Geriatric Disorders, Xiangya Hospital, Central South University, Changsha, China; ^2^Department of Neurology, Xiangya Hospital, Central South University, Changsha, China; ^3^Mobile Health Ministry of Education–China Mobile Joint Laboratory, Xiangya Hospital, Central South University, Changsha, China; ^4^Center for Medical Genetics and Hunan Key Laboratory, School of Life Sciences, Central South University, Changsha, China

**Keywords:** Parkinson’s disease, PD-associated genes, expression pattern, gene expression, age at onset

## Abstract

**Background:**

The expression pattern represents a quantitative phenotype that provides an in-depth view of the molecular mechanism in Parkinson’s disease (PD); however, the expression patterns of PD-associated genes (PAGs) and their relation to age at onset (AAO) remain unclear.

**Methods:**

The known PD-causing genes and PD-risk genes, which were collected from latest published authoritative meta-analysis, were integrated as PAGs. The expression data from Genotype-Tissue Expression database, Allen Brian Map database, and BrainSpan database, were extracted to characterize the tissue specificity, inhibitory-excitatory neuron expression profile, and spatio-temporal expression pattern of PAGs, respectively. The AAO information of PD-causing gene was download from Gene4PD and MDSgene database.

**Results:**

We prioritized 107 PAGs and found that the PAGs were more likely to be expressed in brain-related tissues than non-brain tissues and that more PAGs had higher expression levels in excitatory neurons than inhibitory neurons. In addition, we identified two spatio-temporal expression modules of PAGs in human brain: the first module showed a higher expression level in the adult period than in the prenatal period, and the second module showed the opposite features. It showed that more PAGs belong to the first module that the second module. Furthermore, we found that the median AAO of patients with mutations in PD-causing genes of the first module was lower than that of the second module.

**Conclusion:**

In conclusion, this study provided comprehensive landscape of expression patterns, AAO features and their relationship for the first time, improving the understanding of pathogenesis, and precision medicine in PD.

## Background

Parkinson’s disease (PD) is the second most common neurodegenerative disease (Dorsey et al., 2007; Alzheimer’s Association, 2014), characterized by motor syndromes, such as resting tremor, bradykinesia, rigidity, and postural instability, or non-motor symptoms such as dementia, depression, and sleep disorders (Kalia and Lang, 2015). The prevalence of PD is approximately 1% in the population over the age of 60 years, but reaches up to 5% in individuals older than age 85 years, highlighting the impact of advancing age on the risk of this disease (Ascherio and Schwarzschild, 2016; Lim et al., 2019). The economic burden of PD is up to $23 billion USD per year (Findley, 2007). As life expectancy and aging populations increase worldwide, the number of people with PD is expected to increase by more than 1.5-fold by 2030, making this disease an urgent global health issue (Dorsey et al., 2007).

Although both genetic and environmental factors likely contribute to the development of PD, it shows a high heritability (Blauwendraat et al., 2019a; Li et al., 2019). With the development of high throughput technologies in the last two decades (Jansen et al., 2017; Sandor et al., 2017; Siitonen et al., 2017), thousands of studies have focused on the genetic architectures of PD to understand its pathogenesis (Kalia and Lang, 2015; Labbe et al., 2016; Blauwendraat et al., 2019b). Up to now, genome-wide association studies (GWAS) on the contribution of genetics in a common form of PD (sporadic PD) identified more than 100 loci that were associated with disease risk at a genome-wide significance level, which could sever as potential biomarkers and risk factors for PD (International Parkinson Disease Genomics Consortium, Nalls et al., 2011; Nalls et al., 2014; Chang et al., 2017; Blauwendraat et al., 2019a; Nalls et al., 2019). Additionally, over 20 genes were regarded as PD-causing genes (Blauwendraat et al., 2019b). Meanwhile, we have replicated *PRKN* (Guo et al., 2008), *PINK1* (Tang et al., 2006; Guo et al., 2008), *PARK7* (Tang et al., 2006; Guo et al., 2008), *ATP13A2* (Guo et al., 2008), *PLA2G6* (Shi et al., 2011), *CHCHD2* (Liu et al., 2015), *RAB39B* (Kang et al., 2016), *TMEM230* (Yan et al., 2017), *GCH1* (Xu et al., 2017), and other genes (Tian et al., 2012; Yang et al., 2019) in patients with PD in China. Furthermore, we performed multiplex ligation-dependent probe amplification assays and whole-exome sequencing for 1,676 unrelated patients with PD in a mainland Chinese population. Our data indicate that PD patients with an age at onset (AAO) of 40 years may benefit from genetic counseling, especially those from families with a recessive inheritance pattern (Zhao et al., 2020).

Gene expression studies have been a ubiquitous methodology in biomedical research to deeper understand pathological mechanisms driving diseases (Amar et al., 2015). Meanwhile, gene expression in the patient-personalized medicine may predict the fate of disease, treatment effectiveness, as well as may improve diagnostics and epidemiological disease at a population level (Gire et al., 2014; Fischer et al., 2015; Roychowdhury and Chinnaiyan, 2016; Judge et al., 2020). It is to be noted that gene expression in human brain plays an important role in the development of PD. However, whether PD-associated genes (PAGs) present with a specific expression pattern is unclear. To investigate the specific genetic components of PAGs expression, we systematically characterized the expression pattern of PAGs. This study is expected to provide a comprehensive genetic landscape of PAGs for improving the understanding of the pathogenesis of PD.

## Materials and Methods

The PAGs were integrated with PD-causing genes from a systematic review and PD-risk genes (Blauwendraat et al., 2019b) from iPDGC Locus Browser^1^ (Grenn et al., 2020), which is collecting three GWAS researches (Iwaki et al., 2019; Nalls et al., 2019; Foo et al., 2020). This gene set was further used to investigate the tissue specificity, excitatory-inhibitory neuron expression profile, as well as the spatiotemporal expression pattern.

To explore the tissue specificity of PAGs in different tissues, we sourced the expression table of 54 tissues across 948 donors from the Genotype-Tissue Expression (GTEx)^2^ database (GTEx Consortium et al., 2017). The average expression level of each gene in each tissue was then calculated. For each gene, tissues with the top 50% expression levels in the 53 tissues were defined as preferential expression tissue. In addition, a Reads Per Kilobase per Million mapped reads (RPKM) value of 1 is generally used for defining the threshold of expressed genes. Fisher’s exact test was used to calculate the *P*-value for each tissue.

We analyzed the inhibitory-excitatory neuron expression profile of PAGs based on single-nucleus RNA sequencing data which was downloaded from the Allen Brian Map database^3^. This data set contains 75 transcriptionally distinct cell types from 15,928 nuclei, including 45 GABAergic (inhibitory) neuron types, 24 glutamatergic (excitatory) neuron types, and six non-neuronal types. For each gene in each nucleus, the RPKM value, i.e., the mRNA expression level, was calculated based on the counts per million. Then, the average expression level of each neuronal type for each gene was counted. For the sake of comparison and analysis, we normalized the mean expression values by applying a conversion formula, as follows:

(1) $$\mathrm{Normalized}\,\mathrm{mean}\,\mathrm{expression}\,\mathrm{value}=l\,o\,g_{2}\,\left(e\,x\,p\,r\,e\,s\,s\,i\,o\,n\,m\,e\,a\,n\,v\,a\,l\,u\,e\right)$$

We then used normalized mean expression values to assess the transcriptional profiles in inhibitory and excitatory neurons, and differences in transcription levels were statistically evaluated by the Wilcoxon rank test.

Developmental human brain RNA sequencing data were extracted from the BrainSpan database^4^, which contained 524 tissue-samples of expression data across different developmental stages (from the fetal period to the adult period) and 16 brain regions. To characterize the spatio-temporal expression pattern of PAGs, we performed a weighted gene co-expression network analysis (WGCNA) crossing all tissue samples from BrainSpan to cluster the spatio-temporal expression patterns of PAGs using the standard method with a power of seven. The expression values were normalized by a conversion formula, as follows:

(2) $$N\,o\,r\,m\,a\,l\,i\,z\,e\,d\,m\,e\,a\,n\,e\,x\,p\,r\,e\,s\,s\,i\,o\,n\,v\,a\,l\,u\,e=l\,o\,g_{2}\,\frac{e\,x\,p\,r\,e\,s\,s\,i\,o\,n\,v\,a\,l\,u\,e}{m\,e\,a\,n\,e\,x\,p\,r\,e\,s\,s\,i\,o\,n\,v\,a\,l\,u\,e}$$

In each co-expression module, the temporal-pattern was divided into 12 time-nodes (six before birth time-nodes and six after birth time-nodes); whereas the spatio-pattern was divided into 16 different brain districts, including the hippocampus, amygdala, striatum, mediodorsal nucleus of thalamus, cerebellar cortex, and 11 cerebral cortical districts.

### Association Between Age at Onset and Expression Pattern

The AAO information of each PD-causing genes were collected from MDSgene database (Lill et al., 2016)^5^. We characterized the association of spatio-temporal expression patterns and AAO by comparing the median AAO of genes in a different module, which was achieved by performing a WGCNA. All genes were classified into one of three sections: juvenile-onset (≤30 years), early onset (30–50 years), or late-onset (>50 years), according to the median of the AAO. Then we compared genes with more than five AAO terms to assess the association between AAO and PD-causing genes. After that, we check the association of spatio-temporal expression patterns and AAO by analyzing the other AAO data from Gene4PD^6^.

## Results

In total, we sourced 21 known PD-casuing genes (Blauwendraat et al., 2019b) and 89 PD risk genes (Table 1) from iPDGC Locus Browser^7^ (Grenn et al., 2020), which is collecting three GWAS researches (Iwaki et al., 2019; Nalls et al., 2019; Foo et al., 2020). We noted that three PD-causing genes, such as *LRRK2, SNCA*, and *VPS13C*, were also regarded as PD-risk genes, the other genes in which have suggestive genetic evidence that confer risk to PD from GWAS. Totally, 107 PAGs were collected in this study.

**TABLE 1:** PD associated genes.

PD-causing genes^#^	PD-risk genes^##^
***SNCA***, *PRKN*,	*ADRA2A, ASXL3, BAG3, BIN3, BRIP1, BST1, C5orf24, CAB39L*,
*UCHL1, PARK7*	*CAMK2D, CASC16, CD19, CHD9, CHRNB1, CLCN3, CRHR1, CRLS1*,
***LRRK2**, PINK1*,	*CTSB, DLG2, DNAH17, DYRK1A, ELOVL7, FAM171A2, FAM47E*,
*POLG, HTRA2*,	*FAM49B, FBRSL1, FCGR2A, FGF20, FYN, GAK, GALC, GBAP1, GBF1*,
*ATP13A2, FBXO7*,	*GCH1, GPNMB, GS1-124K5.11, HIP1R, HLA-DRB5, IGSF9B, INPP5F*,
*GIGYF2, GBA*,	*IP6K2, ITGA8, ITPKB, KCNIP3, KCNS3, KPNA1, KRTCAP2, LCORL*,
*PLA2G6, EIF4G1*,	*LINC00693, LOC100131289,**LRRK2**, MAP4K4, MBNL2, MCCC1*,
*VPS35, DNAJC6*,	*MED12L, MEX3C, MIPOL1, NOD2, NUCKS1, PAM, PMVK, RAB29*,
*SYNJ1, DNAJC13*,	*RETREG3, RIMS1, RIT2, RNF141, RPS12, RPS6KL1, SATB1, SCAF11*,
*TMEM230, **VPS13C***,	*SCARB2, SETD1A, SH3GL2, SIPA1L2, SLC44A1, **SNCA**, SPPL2B*,
*LRP10*	*SPTSSB, STK39, SV2C, SYT17, TMEM163, TMEM175, TRIM40, UBAP2*,
	*UBTF, VAMP4, **VPS13C**, WBSCR17, WNT3*

*^#^Known PD-causing genes [Blauwendraat et al. (2019b). The genetic architecture of Parkinson’s disease. Lancet Neurol].*

*^##^Known PD-risk genes [Grenn et al. (2020). The Parkinson’s Disease Genome-Wide Association Study Locus Browser. Mov Disord].*

*Three genes in bold terms, such as SNCA, LRRK2, VPS13C, are regarded as both PD-causing genes and PD-risk genes.*

### PAGs Presented Specific Expression Patterns

**FIGURE 1:**
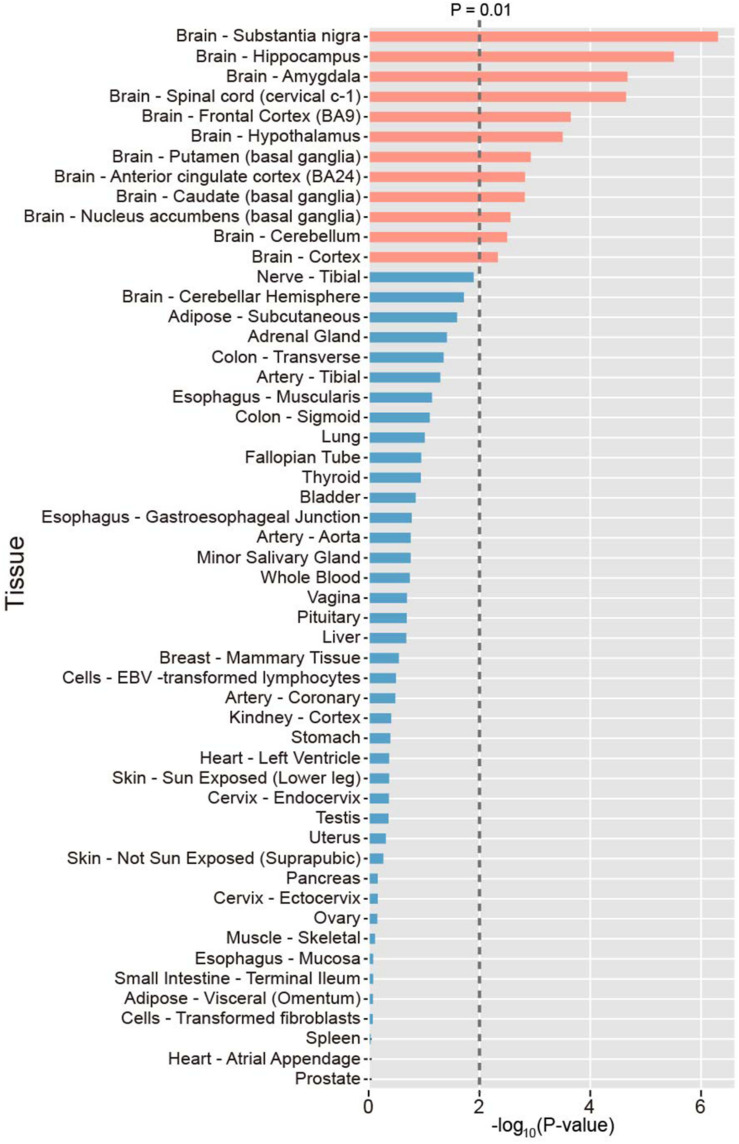
Distribution of the preferential expression tissue of the Parkinson’s disease-associated genes. Tissues with top 50% expression levels were defined the preferential expression tissue. The average expression level in 53 tissues were sourced. *P*-values were calculated by Fisher’s test.

To investigate the tissue specificity of PAGs, we calculated the preferential expression of tissues of each gene and systematically tested the enrichment of preferential expression of tissues of the 107 PAGs using expression data derived from the GTEx project. As a result, we found that the 107 PAGs were significantly expressed in 12 tissues (*P* < 0.01), all of which belong to the brain, including the substantia nigra, hippocampus, amygdala, spinal cord, frontal cortex, hypothalamus, putamen, anterior cingulate cortex, caudate, nucleus accumbens, and cerebellum (Figure 1). This result provides novel evidence that PAGs are likely involved in the other brain specific gene regulation, as well as the known brain-specific gene regulation.

**FIGURE 2:**
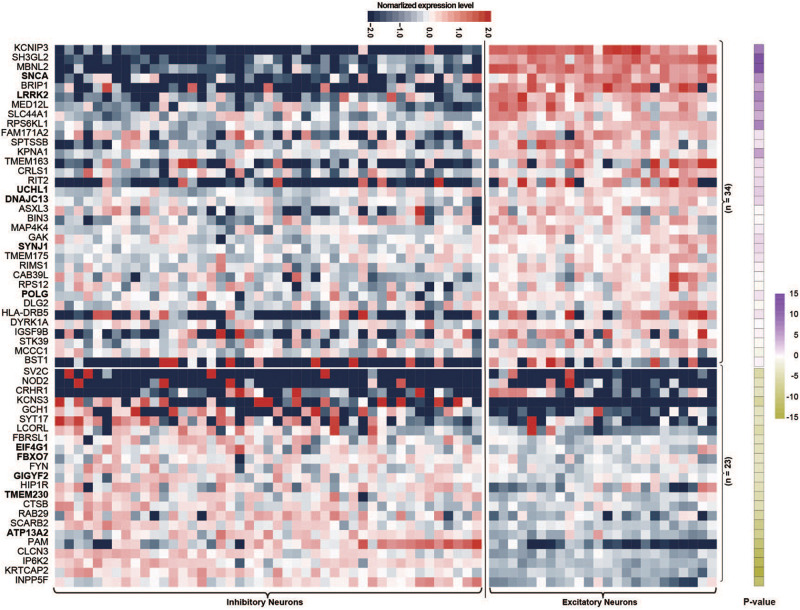
Heat map of normalized expression levels of Parkinson’s disease-associated genes in 45 types of inhibitory neurons and 24 types of excitatory neurons. Only Parkinson’s disease-associated genes showing different expression levels between inhibitory and excitatory neurons are shown; *P*-values were calculated by the Wilcoxon rank sum test.

Single-nucleus RNA sequencing is a good method for evaluating the biological features of genes in neuronal subtypes. In this study, we investigated the expression levels of PAGs in 45 inhibitory neuron types and 24 excitatory neuron types based on The Allen Human Brain Reference Atlas database. For the 107 PAGs, 50 genes did not exhibit significantly different expression levels between excitatory and inhibitory neurons, whereas the other 57 genes presented significant differences in the expression level in the two type of neurons (Supplementary Table 1). Specifically, 34 of the 57 genes (59.65%) including six PD-causing genes (*LRRK2*, *SNCA*, *UCH1*, *DNAJC13*, *SYNJ1, POLG*) had higher expression levels in excitatory neurons than in inhibitory neurons (Figure 2), whereas the other 23 genes (40.35%) including five PD-causing genes (*ATP13A2*, *TMEM230*, *EIF4G1*, *GIGYF2*, *FBXO7*) exhibited the opposite features (Figure 2). This result highlights the essential role of excitatory neurons in the cortical circuit in PD; more functional validation is needed in future studies.

**FIGURE 3:**
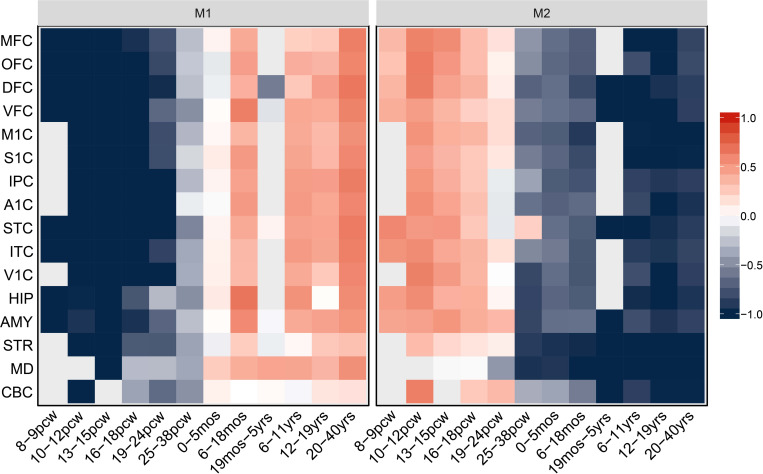
Heat map of normalized expression levels of two spatiotemporal co-expression modules (M1 and M2) corresponding to 17 developmental stages in the human brain. pcw, post-conceptual weeks; mos, moths; yrs, years. All expression levels were normalized according to the log_2_-fold change to the average expression level of each gene.

To further characterize the spatiotemporal expression patterns of the 107 PAGs during brain development from the fetal period to the adult period, we performed WGCNA in brain tissue samples from the BrainSpan database and detected two independent co-expression modules comprising 69 PAGs (Supplementary Table 1). The first module (M1) integrated genes showing low expression in the human embryonic phase which gradually increased in expression starting in the prenatal period (16–18 post-conceptual weeks), and reached a highly stable expression level after birth (both in the adolescent and adult period) (Figure 3). The M1 contained 47 genes, 10 of which are known PD-causing genes (*LRRK2, SNCA, SYNJ1, TMEM230, FBXO7, PINK1, PLA2G6, DNAJC6, GBA, PRKN*) (Supplementary Figure 1). In addition, the second module, which integrated genes highly expressed during the embryonic and early to-middle fetal periods (8–24 post-conceptual weeks), but showed decreased expression starting in the prenatal period and adult period (Figure 3), contained 22 genes including two known PD-causing genes (*VPS35*, *GIGYF2*) (Supplementary Figure 2). Since PAGs were preferentially expressed in the postnatal period compared to the prenatal period, this suggests that genes with enriched postnatal expression are more likely to be associated with PD.

### AAO Is Associated With the Expression Patterns

**FIGURE 4:**
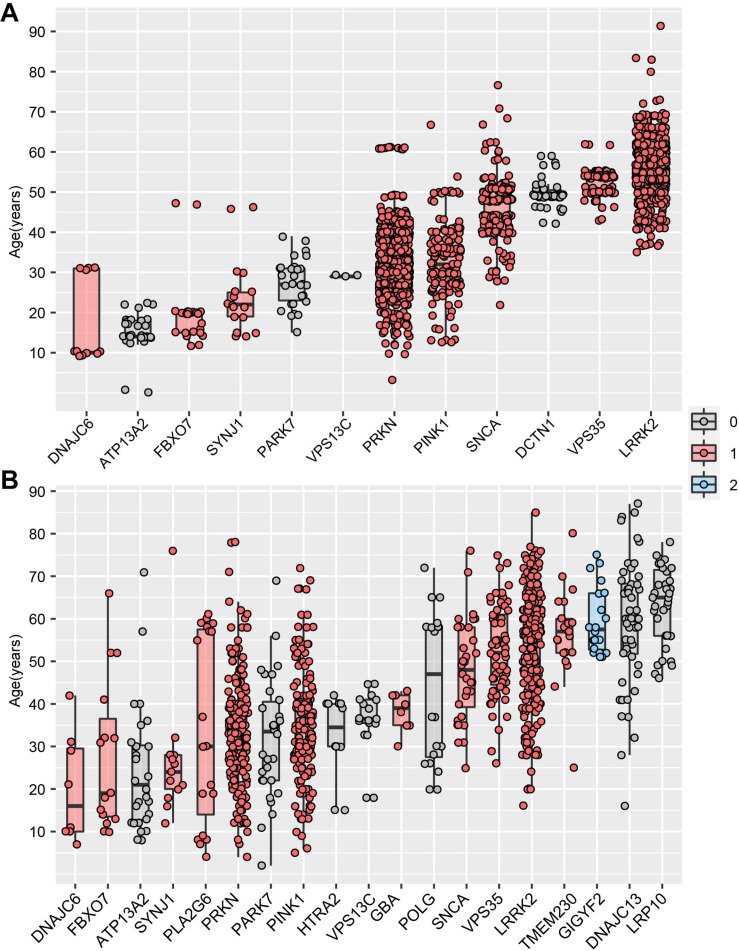
Association between Parkinson’s disease-causing genes and age at onset. **(A)** The association between age at onset (AAO) and Parkinson’s disease-causing genes based on MDSgene database. **(B)** The association between age at onset (AAO) and Parkinson’s disease-causing genes based on Gene4PD database. The genes shown are sorted according to the median AAO or each gene. Genes belonging to spatiotemporal expression module M1 and M2 are highlighted in red and blue, respectively. Genes in gray-filled boxes could not be categorized into either M1 or M2.

MDSgene database (Lill et al., 2016) (see text footnote 5) integrated AAO data and genetic data of 12 PD-causing genes, providing an unprecedented opportunity to identify the vital association between the AAO and PD-causing genes on a large scale. By analyzing the PD-causing genes with more than five AAO items, we found that five genes (*DNAJC6, ATP13A2, FBXO7, SYNJ1, PARK7*) were associated with a juvenile-onset, four genes (*PARKN, PINK1, SNCA, DCTN1*) were associated with an early onset, while *VPS35* and *LRRK2* were associated with a late-onset (Figure 4A). Interestingly, we found that the PD-causing genes in MDSgene were associated with specific spatiotemporal expression pattern (Figure 4A). Specifically, nine of the 12 reported genes in MDSgene belonged to M1, while none of them belonged to M2, and the remaining three genes did not belong to either modules.

We then replicate above results by analyzing the AAO data and genetic data from Gene4PD database^8^. Based on the 21 PD-causing genes reported in the latest review (Blauwendraat et al., 2019b), we analyzed 19 PD-causing genes with more than 5 AAO terns in Gene4PD database. As a result, we found that five of the 19 PD-causing genes were associated with a juvenile-onset, eight genes were associated with an early onset, whereas another six genes were associated with a late-onset (Figure 4B). Meanwhile, 11 of the 19 genes belonged to M1, only *GIGYF2* one gene belonged to M2, and seven genes did not belong to either module. Furthermore, the median AAO of patients with rare variants in *GIGYF2* genes, which is belong to M2, is higher than other genes belong to M1 (Figure 4B). This results above provided novel insight the association among AAO, PD-causing genes, and spatiotemporal expression patterns.

## Discussion

With the development of biotechnology, numerous candidate genes associated with PD have been reported from thousands of published studies. The ability to comprehensively investigate these PAGs from expression patterns in human, would be helpful for studying the pathogenesis of PD (Blauwendraat et al., 2019b).

Analysis of the spatiotemporal gene expression pattern is a widely accepted method for evaluating cellular function at the transcription level in a specific tissue (Li et al., 2017). In this study, we comprehensively analyzed the expression pattern of PAGs with a novel landscape, and many meaningful results were achieved. First, we demonstrated that PAGs were more likely to have higher expression levels in brain tissues than in other tissues. Second, we found that most PAGs had broad expression profiles in excitatory neurons rather than in inhibitory neurons, suggesting that excitatory neurons are more likely associated with PD (Zhang et al., 2016). Furthermore, an interesting study, focused on analyzing the specific gene expression pattern in different cell types associated with PD risk and described a single-cell atlas in human substantia nigra and cortex (Agarwal et al., 2020). According to this atlas, we investigated whether the PAGs were enriched in any cell types. For cortex, it showed that the PAGs were enriched in excitatory neurons (*P* = 1.18e-05) and other four cell types, including astrocyte (*P* = 7.64-e03), microglia (*P* = 0.048), oligodendrocytes (*P* = 0.01), oligo-precursor cells (*P* = 1.78e-03). We noted that the PAGs were also enriched in inhibitory neurons (*P* = 0.07), which is close to be significant (Supplementary Table 2). These results were consisted with our conclusion in this study. For substantia nigra, we also find that the PAGs enriched in Astrocytes (*P* = 4.62e-03), Dopaminergic neurons (*P* = 2.60e-05), GABAergic neurons (*P* = 1.92e-03), Oligodendrocytes (*P* = 2.39e-03), Oligo-precursor cells (*P* = 0.02), but not Endothelial (*P* = 0.15) in substantia nigra (Supplementary Table 2). Notably, the PAGs were most enriched in dopaminergic neurons with the most significant *p*-value. Third, we identified two spatiotemporal expression patterns (M1 and M2) associated with PD. More PAGs belonged to M1 than to M2, highlighting that the high expression level in the adult period is associated with neurodegeneration. However, M2 genes including two PD-causing genes (*VPS35*, *GIGYF2*), were highly expressed during the embryonic and early to-middle fetal period, suggesting that they may associated with neurodevelopment. For example, *GIGYF2* was identified to be associated with macrocephaly (Guo H. et al., 2018). This finding underlines the importance of further research on the mechanisms contributing to the correlation between PD and neurodevelopmental disorders (Hu et al., 2017). Notably, *NUS1*, which belongs to M2, is a novel PD risk gene, which was found to be associated with the early onset of PD in a Han Chinese population in our previous study (Guo J.F. et al., 2018). However, the innovative results in this study were based on the analysis of bioinformatics; thus, the pathogenic mechanisms will need to be verified with cell or animal experiments. Moreover, 47 PAGs including 10 known PAGs in M1, were steadily highly expressed in the adult period (i.e., M1), which is consisted with the feature of adult onset of PD. It can be inferred that other 37 PAGs (Supplementary Table 1), co-expressed with these 10 known PAGs, acting on the same signaling pathway. Our study adds to the evidence that these 37 PAGs contribute to PD. Moreover, another spatiotemporal expression patterns (i.e., M2) which highly expressed during the embryonic and early to-middle fetal period, suggest that not all the PAGs are highly expressed in adult periods, and the pathogenesis of these genes is worth further investigation, which may be helpful to the molecular typing of PD. These hint that the genes, enriched in the postmitotic cells and progenitors or developmental cell states, might involve into different unknown signal ways, respectively. We highly encourage further study to perform more comprehensive analysis with more data.

PD is an age-dependent neurodegenerative condition. The onset age of PD has been widely reported to be related to the variation in phenotypes and genotypes (Wickremaratchi et al., 2011; Kilarski et al., 2012; Nalls et al., 2015). PD-causing genes, such as *ATP13A2* (Ramirez et al., 2006), *PLA2G6* (Paisan-Ruiz et al., 2009), *FBXO7* (Di Fonzo et al., 2009), *PRKN* (Lucking et al., 2000; Djarmati et al., 2004), *PINK1* (Bonifati et al., 2005; Kumazawa et al., 2008), and *PARK7* (Djarmati et al., 2004) have been suggested be strongly associated with AAO. Based on two integrated studies on analyzing the AAO data and genetic data from MDSgene database and Gene4PD database, respectively, we confirmed the association between commonly reported PD-causing genes and AAO. Interestingly, we found that most of the PD-causing genes were belonged to M1, suggesting that the PD-causing genes may be highly expressed after birth. Data from both databases showed very similar trends of each gene, which further examining the relationship between PD-causing genes, AAO and expression pattern.

## Conclusion

In conclusion, we prioritized 107 functionally related PAGs and characterized the genetic landscape of the PAGs by analyzing the expression patterns, associations between AAO and expression pattern. This provided novel insight among the AAO, PAGs, and spatiotemporal expression patterns, as well as provided novel clues for understanding the pathology of PD.

## Data Availability Statement

The original contributions presented in the study are included in the article/Supplementary Material, further inquiries can be directed to the corresponding author/s.

## Author Contributions

BL, BT, and JL: study design. BL, GZ, KL, ZW, ZF, XW, TL, YZ, YW, QC, YH, and JL: literature search and data collection. BL, GZ, KL, ZW, BT, and JL: figures. BL, GZ, ZF, XW, TL, YZ, YW, QC, LD, and JL: data analysis. BL, GZ, YW, QC, JG, BT, and JL: data interpretation. BL, GZ, KL, ZW, ZF, XW, TL, YZ, YW, QC, YH, LD, JG, BT, and JL: writing of the manuscript draft and all revision stages. All authors contributed to the article and approved the submitted version.

## Conflict of Interest

The authors declare that the research was conducted in the absence of any commercial or financial relationships that could be construed as a potential conflict of interest.

## Footnotes

1 https://pdgenetics.shinyapps.io/GWASBrowser/

2 https://gtexportal.org/

3 https://portal.brain-map.org/atlases-and-data/rnaseq

4 http://www.brainspan.org/

5 https://www.mdsgene.org/g4d

6 http://genemed.tech/gene4pd

7 https://pdgenetics.shinyapps.io/GWASBrowser/

8 http://www.genemed.tech/gene4pd

## Abbreviations

PD: Parkinson’s disease
GWAS: genome-wide association studies
PAGs: Parkinson’s disease-associated genes
GTEx: Genotype-Tissue Expression
WGCNA: weighted gene co-expression network analysis.
