# Supplementary material for: Characterizing the Expression Patterns of Parkinson’s Disease Associated Genes
Source: Front Neurosci. 2021 Apr 1;15:629156. doi: 10.3389/fnins.2021.629156 (PMC8049291; doi:10.3389/fnins.2021.629156)
Supplement: Supplementary file 1 [file Table_1.DOCX]

**Characterizing the expression patterns of Parkinson’s disease associated genes**

**SUPPLEMENTAL DATA**

**Table S1. Expression levels in excitatory neurons and inhibitory neurons.**

**Table S2. Enrichment of PD-associated genes in atlas of substantia nigra and cortex**

**Figure S1. The expression patterns of 10 known PD-causing genes in M1.**

**Figure S2. The expression patterns of two known PD-causing genes in M2.**

**Table S1. Expression levels in excitatory neurons and inhibitory neurons.**

| Gene | Inh(mean) | Exc(mean) | P-value | Module |
| --- | --- | --- | --- | --- |
| ADRA2A | 73.22 | 47.94 | 0.81 | 2 |
| ASXL3 | 528.60 | 749.72 | 2.82E-03 | 2 |
| ATP13A2 | 449.49 | 298.86 | 1.92E-06 | 0 |
| BAG3 | 10.66 | 4.33 | 0.16 | 0 |
| BIN3 | 31.54 | 42.82 | 2.55E-03 | 0 |
| BRIP1 | 7.08 | 33.28 | 1.75E-07 | 2 |
| BST1 | 0.91 | 1.06 | 1.98E-03 | 0 |
| C5orf24 | 616.32 | 587.25 | 0.59 | 2 |
| CAB39L | 49.17 | 70.65 | 4.15E-03 | 1 |
| CAMK2D | 358.71 | 543.84 | 0.36 | 1 |
| CD19 | 0.19 | 0.05 | 0.13 | 0 |
| CHD9 | 8231.53 | 8200.01 | 0.85 | 2 |
| CHRNB1 | 10.41 | 12.40 | 0.10 | 0 |
| CLCN3 | 801.69 | 494.11 | 1.72E-10 | 1 |
| CRHR1 | 1.72 | 1.31 | 0.04 | 1 |
| CRLS1 | 61.19 | 89.72 | 1.84E-04 | 2 |
| CTSB | 241.56 | 182.96 | 3.65E-03 | 1 |
| DLG2 | 1216.01 | 1518.77 | 0.02 | 1 |
| DNAH17 | 92.81 | 79.39 | 0.78 | 1 |
| DNAJC13 | 627.52 | 808.03 | 4.21E-05 | 0 |
| DNAJC6 | 883.42 | 838.65 | 0.38 | 1 |
| DYRK1A | 255.60 | 299.97 | 4.15E-03 | 2 |
| EIF4G1 | 206.93 | 169.02 | 0.02 | 0 |
| ELOVL7 | 15.22 | 23.91 | 0.10 | 1 |
| FAM171A2 | 9.40 | 15.73 | 4.72E-04 | 2 |
| FAM47E | 34.68 | 37.43 | 0.58 | 1 |
| FAM49B | 215.69 | 221.24 | 0.53 | 0 |
| FBRSL1 | 119.71 | 96.82 | 0.02 | 0 |
| FBXO7 | 110.72 | 96.44 | 0.05 | 1 |
| FCGR2A | 6.36 | 5.98 | 0.77 | 0 |
| FGF20 | 1.95 | 1.31 | 0.18 | 1 |
| FYN | 150.81 | 125.40 | 0.03 | 2 |
| GAK | 200.58 | 261.57 | 1.10E-04 | 0 |
| GALC | 168.26 | 143.41 | 0.11 | 1 |
| GBA | 21.32 | 19.64 | 0.85 | 1 |
| GBF1 | 250.38 | 259.75 | 0.28 | 0 |
| GCH1 | 15.11 | 5.60 | 0.03 | 1 |
| GIGYF2 | 442.67 | 378.78 | 0.03 | 2 |
| GPNMB | 13.46 | 1.82 | 0.46 | 1 |
| HIP1R | 217.77 | 160.26 | 7.95E-03 | 0 |
| HLA-DRB5 | 0.27 | 0.64 | 5.02E-04 | 1 |
| HTRA2 | 20.15 | 20.77 | 0.56 | 0 |
| IGSF9B | 12.78 | 22.34 | 1.52E-03 | 0 |
| INPP5F | 1685.30 | 927.12 | 1.31E-09 | 1 |
| IP6K2 | 256.56 | 152.45 | 2.03E-11 | 2 |
| ITGA8 | 133.57 | 48.31 | 0.11 | 1 |
| ITPKB | 20.55 | 13.45 | 0.37 | 1 |
| KCNIP3 | 3.75 | 45.34 | 2.05E-11 | 1 |
| KCNS3 | 64.59 | 0.46 | 1.00E-04 | 1 |
| KPNA1 | 295.23 | 418.04 | 4.32E-08 | 2 |
| KRTCAP2 | 51.18 | 30.63 | 7.00E-14 | 0 |
| LCORL | 215.71 | 164.10 | 8.83E-04 | 2 |
| LRP10 | 2.12 | 2.28 | 0.45 | 0 |
| LRRK2 | 432.69 | 1160.45 | 1.03E-10 | 1 |
| MAP4K4 | 611.20 | 797.41 | 9.27E-04 | 2 |
| MBNL2 | 105.04 | 501.39 | 1.73E-18 | 1 |
| MCCC1 | 135.60 | 150.39 | 0.05 | 0 |
| MED12L | 243.50 | 567.91 | 3.92E-09 | 1 |
| MEX3C | 23.80 | 27.27 | 0.28 | 2 |
| MIPOL1 | 123.92 | 135.09 | 0.11 | 2 |
| NOD2 | 0.73 | 0.51 | 9.76E-03 | 0 |
| NUCKS1 | 114.46 | 112.07 | 0.64 | 2 |
| PAM | 1668.75 | 468.12 | 9.03E-09 | 1 |
| PARK7 | 62.54 | 65.45 | 0.40 | 0 |
| PINK1 | 27.49 | 30.58 | 0.10 | 1 |
| PLA2G6 | 13.63 | 16.76 | 0.14 | 1 |
| PMVK | 15.77 | 13.48 | 0.08 | 0 |
| POLG | 107.15 | 128.72 | 3.01E-04 | 0 |
| RAB29 | 18.32 | 10.66 | 7.42E-05 | 1 |
| RIMS1 | 746.54 | 937.75 | 1.24E-03 | 1 |
| RIT2 | 7.04 | 27.65 | 7.65E-05 | 1 |
| RNF141 | 35.05 | 40.21 | 0.22 | 1 |
| RPS12 | 26.18 | 34.54 | 0.01 | 2 |
| RPS6KL1 | 182.83 | 280.74 | 1.33E-10 | 0 |
| SATB1 | 668.75 | 703.39 | 0.09 | 0 |
| SCAF11 | 1043.96 | 1080.96 | 0.57 | 0 |
| SCARB2 | 280.81 | 197.35 | 7.53E-07 | 2 |
| SETD1A | 99.81 | 87.04 | 0.76 | 0 |
| SH3GL2 | 60.46 | 333.46 | 1.73E-18 | 1 |
| SIPA1L2 | 582.15 | 391.22 | 0.16 | 0 |
| SLC44A1 | 270.98 | 485.63 | 2.58E-07 | 1 |
| SNCA | 48.33 | 163.31 | 1.21E-09 | 1 |
| SPPL2B | 55.36 | 48.58 | 0.30 | 1 |
| SPTSSB | 46.46 | 83.18 | 3.07E-04 | 1 |
| STK39 | 96.80 | 124.57 | 0.02 | 1 |
| SV2C | 152.13 | 37.56 | 4.59E-04 | 1 |
| SYNJ1 | 1236.65 | 1558.07 | 1.31E-04 | 1 |
| SYT17 | 185.53 | 107.19 | 0.04 | 1 |
| TMEM163 | 15.17 | 35.85 | 1.07E-04 | 0 |
| TMEM175 | 153.76 | 196.07 | 3.53E-04 | 0 |
| TMEM230 | 81.94 | 64.08 | 2.06E-03 | 1 |
| TRIM40 | 0.08 | 0.01 | 0.89 | 0 |
| UBAP2 | 155.68 | 177.68 | 0.17 | 2 |
| UBTF | 55.39 | 51.44 | 0.97 | 2 |
| UCHL1 | 707.62 | 933.31 | 1.72E-05 | 0 |
| VAMP4 | 38.08 | 39.36 | 0.81 | 1 |
| VPS13C | 9391.89 | 7821.97 | 0.17 | 0 |
| VPS35 | 635.42 | 589.16 | 0.31 | 1 |
| WNT3 | 18.90 | 16.13 | 0.94 | 0 |

**Table S2. The expression pattern enrichment of PD-associated genes in atlas of substantia nigra and cortex***

| Brian regions | Cell types | Number of PD associated genes | Number of genes of PD risk* | P value of enrichment |
| --- | --- | --- | --- | --- |
| Cortex | **Excitatory neurons** | **20** | **1315** | **1.18e-05** |
|  | **Inhibitory neurons** | **7** | **767** | **0.07** |
|  | Astrocyte | 8 | 613 | 7.64-e03 |
|  | Microglia | 3 | 244 | 0.048 |
|  | Oligodendrocytes | 5 | 299 | 0.01 |
|  | Oligo-precursor cells | 5 | 225 | 1.78e-03 |
| Substantia nigra | Astrocytes | 11 | 901 | 4.62e-03 |
|  | Dopaminergic neurons | 21 | 1500 | 2.60e-05 |
|  | Endothelial | 1 | 122 | 0.15 |
|  | GABAergic neurons | 17 | 1521 | 1.92e-03 |
|  | Oligodendrocytes | 7 | 414 | 2.39e-03 |
|  | Oligo-precursor cells | 4 | 264 | 0.02 |

*Agarwal et al. Nature Communications, 2020


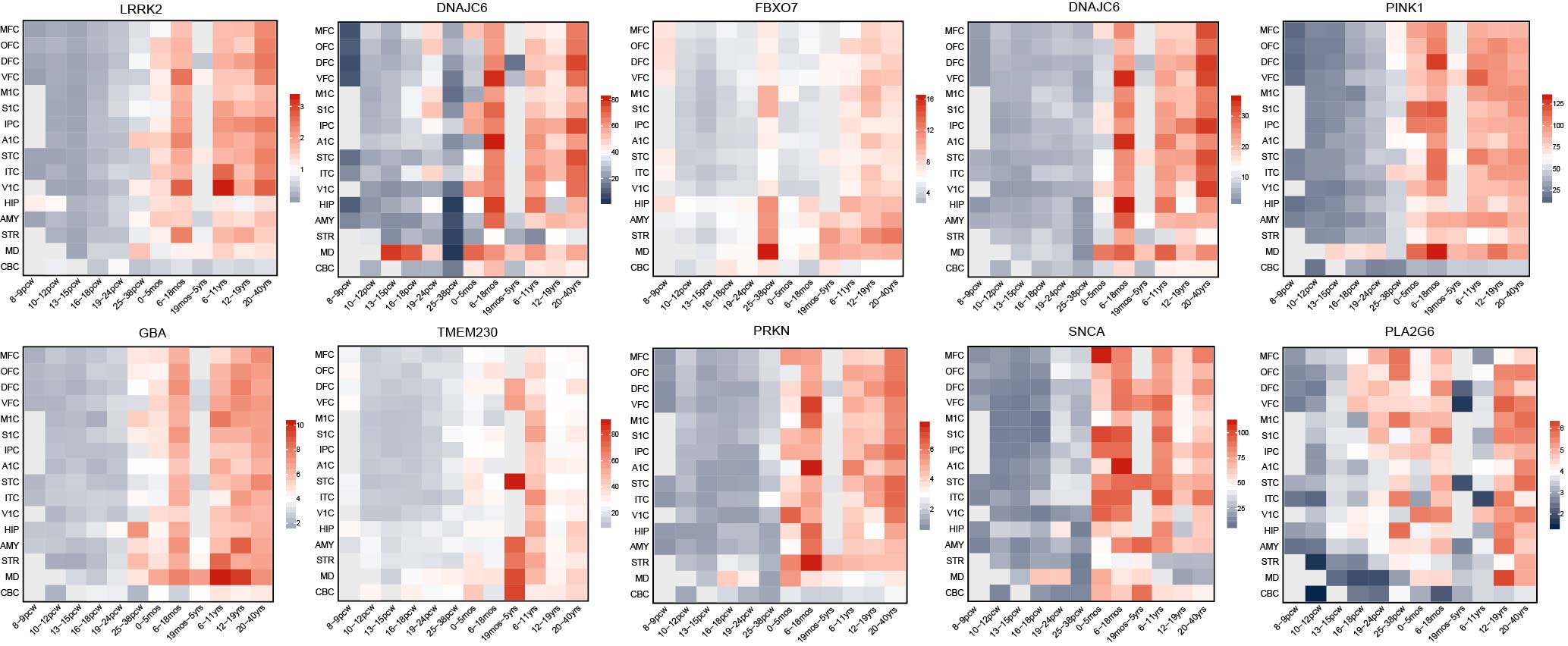
 **Figure S1. The expression patterns of 10 known PD-causing genes in M1.**


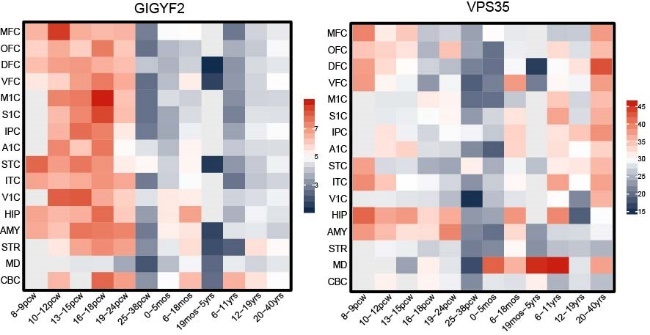


**Figure S2. The expression patterns of two known PD-causing genes in M2.**
